# Age and Sex Influences Gamma-aminobutyric Acid Concentrations in the Developing Brain of Very Premature Infants

**DOI:** 10.1038/s41598-020-67188-y

**Published:** 2020-06-29

**Authors:** Sudeepta K. Basu, Subechhya Pradhan, Marni B. Jacobs, Mariam Said, Kushal Kapse, Jonathan Murnick, Matthew T. Whitehead, Taeun Chang, Adre J. du Plessis, Catherine Limperopoulos

**Affiliations:** 1 Neonatology, Children’s National Hospital, Washington, D.C US; 2 Center for the Developing Brain, Children’s National Hospital, Washington, D.C US; 3 Division of Biostatistics and Study Methodology, Children’s National Hospital, Washington, D.C US; 4 Division of Diagnostic Imaging and Radiology, Children’s National Hospital, Washington, D.C US; 5 Division of Neurology, Children’s National Hospital, Washington, D.C US; 6 Fetal Medicine institute, Children’s National Hospital, Washington, D.C US; 7 The George Washington University School of Medicine, Washington, D.C US

**Keywords:** Diagnostic markers, Neurodevelopmental disorders, Neonatal brain damage

## Abstract

Gamma-aminobutyric acid (GABA) and glutamate are principal neurotransmitters essential for late gestational brain development and may play an important role in prematurity-related brain injury. *In vivo* investigation of GABA in the preterm infant with standard proton magnetic resonance spectroscopy (^1^H-MRS) has been limited due to its low concentrations in the developing brain, and overlap in the spectrum by other dominant metabolites. We describe early postnatal profiles of *in vivo* GABA and glutamate concentrations in the developing preterm brain measured by using the J-difference editing technique, Mescher-Garwood point resolved spectroscopy. We prospectively enrolled very preterm infants born ≤32 weeks gestational age and non-sedated ^1^H-MRS (echo time 68 ms, relaxation time 2000 ms, 256 signal averages) was acquired on a 3 Tesla magnetic resonance imaging scanner from a right frontal lobe voxel. Concentrations of GABA + (with macromolecules) was measured from the J-difference spectra; whereas glutamate and composite glutamate + glutamine (Glx) were measured from the unedited (OFF) spectra and reported in institutional units. We acquired 42 reliable spectra from 38 preterm infants without structural brain injury [median gestational age at birth of 28.0 (IQR 26.0, 28.9) weeks; 19 males (50%)] at a median postmenstrual age of 38.4 (range 33.4 to 46.4) weeks. With advancing post-menstrual age, the concentrations of glutamate OFF increased significantly, adjusted for co-variates (generalized estimating equation β = 0.22, p = 0.02). Advancing postnatal weeks of life at the time of imaging positively correlated with GABA + (β = 0.06, p = 0.02), glutamate OFF (β = 0.11, p = 0.02) and Glx OFF (β = 0.12, p = 0.04). Male infants had higher GABA + (1.66 ± 0.07 vs. 1.33 ± 0.11, p = 0.01) concentrations compared with female infants. For the first time, we report the early ex-utero developmental profile of *in vivo* GABA and glutamate stratified by age and sex in the developing brain of very preterm infants. This data may provide novel insights into the pathophysiology of neurodevelopmental disabilities reported in preterm infants even in the absence of structural brain injury.

## Introduction

Advances in perinatal intensive care have improved survival and decreased severe sensory-motor impairments and neuro-developmental disabilities in very preterm infants [born before 32 weeks gestational age (GA)]^1–3^. Despite these important advances, 1 in 3 surviving very preterm infants continue to suffer from debilitating cognitive and social-behavioral impairments even in the absence of destructive brain lesions such as high-grade intraventricular hemorrhage or periventricular leukomalacia^4,5^. The importance of early detection of subtle brain injury is emphasized by the increasing population of surviving preterm infants with special needs^2,5^. These data suggests that less fulminant microstructural brain injury or disturbed maturation, undetected by head ultrasound and conventional magnetic resonance imaging (MRI), may occur during the rapid phase of late gestational development^6^. Mid-to-late gestational brain development includes neurogenesis, migration, dendrite arborization and synaptogenesis which are heavily dependent on gamma-aminobutyric acid (GABA) and glutamatergic neural systems and are at risk of disruption in the hostile extra-uterine environment after preterm birth^7–9^. *Ex-vivo* studies of the preterm brain have demonstrated specific loss of GABA-ergic neuron populations, alteration of GABA receptor subunits, disorganized migration and neuronal differentiation^10–12^. Assessment of GABA and glutamate in the developing human brain would need a non-invasive tool that can be applied reliably in the critically-ill preterm infant, often dependent on intensive care support during early postnatal life.

Proton magnetic resonance spectroscopy (^1^H-MRS) enables non-invasive measurement of the *in vivo* concentrations of neurometabolites like N-acetyl-aspartate (NAA), choline (Cho) and creatine (Cr); which have been associated with neurodevelopmental outcomes^13^. GABA and glutamate signals in the standard ^1^H-MRS spectrum on conventional 1.5 Tesla MRI are overlapped by other dominant metabolites, which necessitate using higher field strength MR scanners, shorter echo-times or spectral editing techniques to improve their signal resolution^14,15^. Studies using the spectral J-difference editing technique, MEscher-GArwood Point Resolved Spectroscopy (MEGA-PRESS) have reported lower regional GABA and glutamate concentrations in older subjects with disorders like epilepsy, autism, attention-deficit disorder^14,16^, known to affect a high proportion of prematurely born surviving adults^3^. While interpreting these results, it should be noted that measurements by MEGA-PRESS have contributions from other co-edited metabolites; e.g macromolecules with GABA (represented as GABA+) and glutamine with glutamate (represented as Glx)^14,15^. However, *in vivo* assessment of GABA in the developing human preterm brain has been limited by motion artifacts on non-sedated neonatal scans, the small brain volumes limiting the size of the ^1^H-MRS voxel, the limited availability of high magnetic field strength MRI scanners for this population and additional challenges associated with intensive care support during early postnatal life. Recent reports using the MEGA-PRESS technique have shown lower GABA and glutamate concentrations at term-equivalent age (TEA, between 37 to 41 weeks postmenstrual age) and a negative correlation with functional connectivity in small cohorts of preterm infants (born <35 weeks gestational age) compared to healthy full-term infants^17–19^.

In this study, we acquired MEGA-PRESS spectra to simultaneously characterize the temporal profiles of *in vivo* brain GABA and glutamate concentrations in the developing frontal lobe of very preterm infants during the ex-utero third trimester and at term equivalent age (TEA). We hypothesized that GABA and glutamate concentrations in the developing frontal lobe would increase with advancing postmenstrual age (PMA) in very preterm infants.

## Methods

### Participants

Very premature infants born at ≤32 weeks GA and birth weight ≤1500 g, admitted in the neonatal intensive care unit (NICU) at Children’s National Hospital (Washington, D.C.) between 2016 and 2018 were prospectively enrolled. Infants with congenital malformations or dysmorphic features, confirmed metabolic disorder, genetic syndrome or chromosomal abnormality were excluded. For this report, only infants without significant structural brain injury on T2-weighted images on MRI identified by Kidokoro score were included for analysis. Demographic, perinatal and postnatal clinical data were collected through medical records review and parental questionnaires. The study was approved by the Children’s National Hospital Institutional Review Board and conducted in accordance with relevant guidelines and regulations. Written informed consent was obtained from the parent(s) of each participant.

### MRI & ^1^H-MRS acquisition

Enrolled infants underwent an MRI after reaching TEA (PMA ≥ 38 weeks) on a 3 Tesla MR scanner (Discovery MR750, General Electric Medical Systems, Waukesha, WI). A subset of the cohort also underwent an early postnatal MRI on the 3-T scanner when medically stable before 37 weeks PMA. All preterm and TEA ^1^H-MRS meeting the quality standards are reported cross-sectionally and adjusted for repeated measures using generalized estimating equations. MRIs were performed during natural sleep using a feed and swaddle technique and unless clinically indicated, sedatives were not administered during the MRI. Preterm infants requiring temperature monitoring were scanned using an MRI-compatible incubator (LMT Medical Systems GmbH, Luebeck, Germany) and eight-channel incubator LMT receiver head coil.

**Figure 1:**
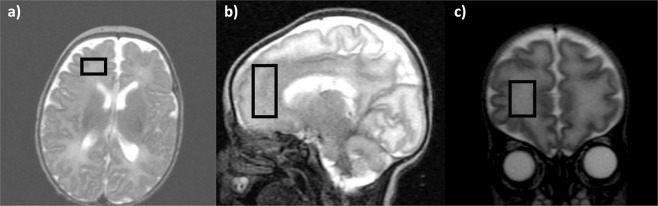
^1^H -MRS voxel placement in the preterm right frontal lobe: (**a**) Axial, (**b**) Sagittal and (**c**) Coronal views.

**Figure 2:**
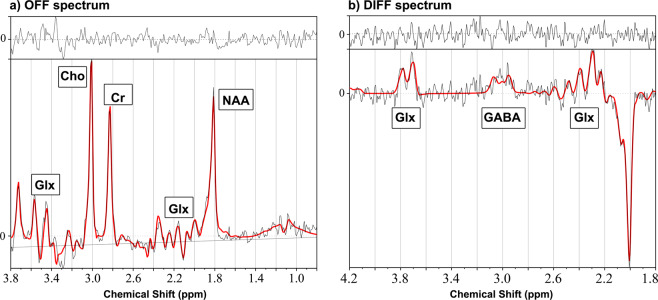
Representative ^1^H-MRS LCModel spectral output for (**a**) OFF and (**b**) DIFF MEGA-PRESS in a preterm infant.

^1^H-MRS was acquired from a 20 mm ×15 mm ×15 mm voxel (2.7 cm^3^ average volume) placed in the right frontal lobe sub-cortical white matter (Fig. 1). MEGA-PRESS sequence with a TE of 68 ms, a TR of 2000 ms, spectral width of 5000 Hz with 4096 points and 256 signal averages were acquired by placing the editing pulses at 1.9 ppm during ‘ON’ and 7.5 ppm during ‘OFF’ acquisitions, respectively. J-difference (DIFF) spectrum was generated by subtracting the ‘OFF’ spectrum from the ‘ON’ spectrum (representative DIFF and OFF spectrum Fig. 2 a & b; composite all OFF and DIFF spectra Supplement Fig. A & B). Frequency selective amplitude modulated pulses of 16 ms duration were used as editing pulses in our MEGA-PRESS acquisitions. Eight averages of water unsuppressed spectra were acquired for use as a reference peak for the analysis.

### Brain injury classification from structural MRI

T2- weighted brain images from each MRI study at TEA was reviewed and scored using the Kidokoro scoring system by an experienced pediatric neuroradiologist (JM) to identify the presence of moderate-to-severe brain injury (score of ≥2 for cerebellar signal abnormality and/or score of ≥2 for cystic, signal or myelination abnormality in either cerebral WM, cortical GM or deep GM)^20^. Infants with structural brain injury based on this classification were excluded from the analysis for this report.

### 1H-MRS Data Pre- and Post-processing

^1^H-MRS data were frequency and phase-corrected using a previously described method^21,22^ and the resulting ‘OFF’ and ‘ON’ spectra were used to generate the DIFF spectrum. Two sets of spectra OFF acquisition and DIFF spectra for each dataset were analyzed using LCModel to measure metabolite concentrations, using the unsuppressed water signal as an internal reference.^23^,^24^. The basis set used for LCModel analysis was received from Purdue University (http://purcell.healthsciences.purdue.edu/mrslab/basis_sets.html). The OFF basis set included 14 metabolites: alanine, aspartate, creatine, GABA, glutamate, glutamine, glutathione, glycerophosphorylcholine (GPC), lactate, myo-inositol, N-acetylaspartate (NAA), N-acetylaspartyl glutamate (NAAG), scyllo-inositol, and taurine. J-difference basis set included the following 6 metabolites: GABA, glutamine, glutamate, glutathione, NAA and NAAG. The LCModel outputs were first visually screened for artifacts. A representative spectrum with fit results for the DIFF and OFF spectra are shown in Fig. 2 a & b respectively. From the LCModel output, we used full width at half maximum (FWHM) of <11 Hz as inclusion criteria for the spectral quality. Further, we used signal-to-noise ratio (SNR) ≥ 5 cut-off for the OFF spectra and SNR ≥ 2 cut-off for the DIFF spectra.

GABA measurements from DIFF MEGA-PRESS at 1.9 ppm include contribution from co-edited resonances from unspecified macromolecule resonance and hence represented as GABA + ^15^. Concentrations of glutamate and Glx (glutamate + glutamine), NAA (N-Acetylaspartate + N-acetyl-aspartyl-glutamate), Cho (glycerophosphorylcholine + phosphocholine), Cr (creatine + phosphocreatine) and Ins (inositol) were measured from the OFF spectra from the LCModel output. Glutamate and glutamine also co-edits during MEGA-PRESS editing for GABA and the resulting resolved peaks around 2.0–2.2 ppm allowed simultaneous measurement in LCModel, represented as glutamate DIFF and Glx DIFF in our results^25^. We also investigated the correlation between the measured glutamate and Glx concentrations from the OFF and DIFF spectra^25^. All metabolite concentrations are reported in institutional units (i.u.). Given the low GABA, glutamate and Glx levels in the preterm brain, we accepted Cramer-Rao lower bound (CRLB) confidence intervals up to 40% for these metabolites to obtain a more representative data sample^13,19,26^. All other metabolite concentrations were included for analysis only if their CRLB were lower than 20%. These inclusion parameters based on CRLB cut-offs, were adopted a priori, consistent with our previous publication^27^.

### Statistical analysis

Wilcoxon Mann-Whitney and Fisher’s exact tests, as appropriate, were used for descriptive analyses to compare medians and frequencies of demographic and clinical characteristics by sex (male vs female) and GA at birth (<28 weeks vs ≥28 weeks; post-hoc analysis). Since a small number of participants had ^1^H-MRS metabolite measurements at multiple time-points, mean metabolite levels overall with standard errors were estimated using Taylor series variance estimation techniques allowing for clustering at the patient level. Metabolite concentrations were compared across sex and categorized gestational age at birth using least squares means estimates from generalized estimating equations (GEE) adjusted for within patient clustering. GEE models, which adjust for correlations between multiple measures of the same infant, were subsequently used to investigate the relationship between infant’s PMA and sex and metabolite concentrations using Type 3 score statistics. To investigate the influence of duration of in-utero gestation (GA at birth) and postnatal ex-utero (weeks of life, WOL) development on the metabolite concentrations, we included them as distinct age parameters in GEE regression models. Bivariate associations between metabolites and potential covariates were considered; those associated with any metabolite at p < 0.05 were included in multivariable GEE models to control for any confounding effects. Analyses were conducted using SAS 9.4 (SAS Institute Inc, Cary NC, USA). A 2-tailed alpha level of 0.05 was considered statistically significant.

## Results

### Descriptive Characteristics of our cohort

We acquired ^1^H-MRS spectra from 38 preterm infants [median 28.0 (IQR 26.0, 28.9) weeks GA at birth; median birth weight 978 (750, 1198) grams and 19 males (50%)] at a median PMA of 38.4 (range 33.4 to 46.4) weeks (Table 1). Four infants had reliable preterm as well as TEA ^1^H-MRS spectra accounting for a total of 42 analyzed spectra. Demographic characteristics are presented in Table 1 and were similarly distributed among male and female infants. Only one infant received clinically indicated weaning dose morphine on the day of ^1^H-MRS acquisition. Metabolite concentrations and ratios in the overall cohort and stratified by sex are reported in Table 1 using the CRLB inclusion cut-offs described in Methods section. In the supplementary Table A, we have also reported GABA and glutamate concentrations using a range of CRLB cut-offs.

**Table 1:** Baseline characteristics of preterm infants and metabolite concentrations stratified by sex of the infant.

Infant parameters	Infants with Reliable ^1^H –MRS (n = 38)	Male infants (n = 19)	Female infants (n = 19)	p-value^a^
**GA at birth in weeks (median, IQR)**	28.0 (26.0, 28.9)	26.9 (26.0, 29.0)	28.0 (26.0, 28.7)	0.77
**Birth weight (median, IQR)**	978 (750, 1198)	925 (720, 1015)	1080 (767, 1280)	0.24
**Small-for-gestation infants (n, %)**	4 (10.5)	2 (10.5)	2 (10.5)	1.00
**Male Sex (n, %)**	19 (50.0)			
**African American (n, %)**	24 (63.2)	14 (73.7)	10 (52.6)	0.31
**Cesarean Section delivery (n, %)**	25 (65.8)	12 (63.2)	13 (68.4)	1.00
**Multiple gestation (n, %)**	8 (21.1)	5 (26.3)	3 (15.8)	0.69
**Apgar at 5 min (median, IQR)**	8 (7, 8)	8 (7, 8)	8 (7, 8)	0.36
**PMA at** ^**1**^**H–MRS in weeks (median, IQR)**	38.4 (37.1, 40.6)	38.7 (37.7, 41.9)	38.2 (37.0, 39.9)	0.34
**Weeks of life at** ^**1**^**H–MRS (median, IQR)**	11.2 (9.3, 13.7)	11.5 (9.3, 14.1)	10.6 (8.3, 13.1)	0.23
Metabolites (mean ± SE in institutional units), accounting for patient clustering; (n = number of infants with reliable measurements)				
**GABA** + **(n** = **35)**	1.50 ± 0.07	1.66 ± 0.07	1.33 ± 0.11	**0.01**
**Glutamate DIFF (n** = **33)**	2.64 ± 0.15	2.82 ± 0.18	2.44 ± 0.23	0.20
**Glutamate OFF (n** = **19)**	3.09 ± 0.20	3.19 ± 0.24	2.92 ± 0.31	0.50
**Glx DIFF (n** = **36)**	3.84 ± 0.27	4.54 ± 0.40	3.07 ± 0.26	**0.002**
**Glx OFF (n** = **33)**	4.43 ± 0.19	4.66 ± 0.24	4.18 ± 0.28	0.19
**NAA (n** = **38)**	3.71 ± 0.19	3.95 ± 0.28	3.46 ± 0.24	0.18
**Choline (n** = **38)**	1.99 ± 0.05	2.08 ± 0.05	1.90 ± 0.07	**0.04**
**Cr (n** = **38)**	3.54 ± 0.11	3.73 ± 0.15	3.34 ± 0.14	0.06
**Ins (n** = **38)**	6.77 ± 0.22	6.93 ± 0.30	6.59 ± 0.29	0.41
**GABA** +**/Cho (n** = **35)**	0.74 ± 0.03	0.79 ± 0.03	0.68 ± 0.06	0.10
**GABA** +**/Cr (n** = **35)**	0.42 ± 0.02	0.44 ± 0.02	0.39 ± 0.04	0.20
**Glutamate DIFF/Cho (n** = **33)**	1.31 ± 0.07	1.36 ± 0.08	1.26 ± 0.11	0.47
**Glutamate DIFF/Cr (n** = **33)**	0.73 ± 0.03	0.75 ± 0.03	0.71 ± 0.06	0.62
**NAA/Cr (n** = **38)**	1.03 ± 0.03	1.04 ± 0.04	1.03 ± 0.04	0.82
**NAA/Cho (n** = **38)**	1.85 ± 0.08	1.90 ± 0.12	1.80 ± 0.08	0.54
**Cr/Cho (n** = **38)**	1.79 ± 0.04	1.82 ± 0.06	1.76 ± 0.04	0.40
**DIFF SNR (median, IQR)**	5 (4, 7)	6 (4, 8)	5 (4, 6)	0.40
**OFF SNR (median, IQR)**	9 (7, 10)	9 (7, 10)	8 (7, 10)	0.31
**DIFF FWHM (median, IQR)**	0.06 (0.04, 0.08)	0.06 (0.04, 0.07)	0.07 (0.05, 0.10)	0.12
**OFF FWHM (median, IQR)**	0.04 (0.03, 0.05)	0.04 (0.03, 0.05)	0.05 (0.03, 0.05)	0.34

SNR, signal-to-noise ratio; FWHM, full width at half-maximum.

All metabolite concentrations are in institutional units.

^a^Wilcoxon Mann-Whitney and Fisher’s exact test for demographic and clinical comparisons between male and female infants; least squares means estimates from GEE models for comparison of metabolite concentrations between male and female infants.

### Relationship between GABA + and glutamate concentrations with advancing age

**Figure 3:**
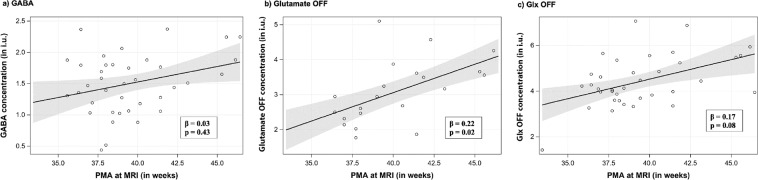
Metabolite profiles with advancing PMA at MRI: (**a**) GABA (**b**) Glutamate OFF (**c**) Glx OFF.

The associations between metabolite concentrations and ratios with GA at birth, PMA at MRI and postnatal WOL at MRI are reported in Table 2. With advancing PMA, we observed a significant increase in concentrations of glutamate OFF (generalized estimating equation regression β = 0.22, p = 0.02, Fig. 3b) NAA (β = 0.31, p = 0.006, Supplementary Fig. C) and Cr (β = 0.14, p = 0.01) adjusted for co-variates (GA at birth, mode of delivery, infant’s sex, race and Apgar score at 5 minutes of life); but not for GABA + (β = 0.03, p = 0.43 Fig. 3a). Advancing postnatal WOL at ^1^H-MRS correlated positively with most metabolite concentrations including GABA + (β = 0.06, p = 0.02), glutamate OFF (β = 0.11, p = 0.02) and Glx OFF (β = 0.12, p = 0.04) (Table 2). We observed an unadjusted decrease in concentrations of GABA + (β = −0.08, p = 0.01) with increasing GA at birth (Table 2).

**Table 2:** Relationship of right frontal lobe metabolite concentrations with infant’s age (postmenstrual, gestational and postnatal), and sex.

Metabolite	Association between metabolites and infant’s age and sex (β, p-value)				
	Postmenstrual age ^1^H–MRS	Gestational Age at birth	Postnatal age at ^1^H–MRS	Male sex	PMA at ^1^H–MRS^a^
GABA +	0.05, 0.11	−0.08, **0.01**	0.06, **0.02**	0.33, **0.02**	0.03, 0.43
Glutamate DIFF	0.14, **0.02**	−0.15, 0.06	0.13, **0.005**	0.37, 0.21	0.12, 0.07
Glutamate OFF	0.16, **0.007**	−0.06, 0.55	0.11, **0.02**	0.27, 0.50	0.22, **0.02**
Glx DIFF	0.25, **0.01**	−0.20, **0.04**	0.20, **0.008**	1.46, **0.005**	0.17, **0.02**
Glx OFF	0.17, **0.03**	−0.08, 0.37	0.12, **0.04**	0.48, 0.20	0.17, 0.08
NAA	0.32, **0.002**	−0.10, 0.20	0.21, **0.003**	0.49, 0.19	0.31, **0.006**
Choline	0.03, 0.08	−0.05, **0.03**	0.03, **0.03**	0.18, 0.05	0.02, 0.36
Cr	0.18, **0.003**	−0.08, 0.20	0.13, **0.008**	0.39, 0.07	0.14, **0.01**
Ins	0.09, 0.29	−0.10, 0.32	0.08, 0.22	0.34, 0.42	0.06, 0.38
GABA +/Cho	0.01, 0.27	−0.02, **0.05**	0.02, 0.09	0.11, 0.11	0.01, 0.57
GABA +/Cr	−0.01, 0.28	−0.02, **0.04**	0.00, 0.79	0.05, 0.21	−0.01, 0.27

Estimates from generalized estimating equations.

^a^Adjusted for GA at birth, sex, race, and 5 minute Apgar.

Both NAA and Cr concentrations increased significantly with advancing PMA and postnatal WOL at MRI, but not GA at birth. Cho and Ins concentrations were not significantly associated with PMA or sex (Table 2).

### Relationship of GABA + concentrations with sex

**Figure 4:**
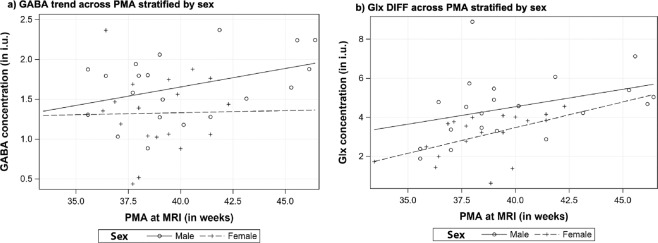
Temporal profile of (**a**) GABA and (**b**) Glx DIFF with advancing PMA at MRI stratified by infant’s sex.

Male infants had higher concentrations of GABA + (1.66 ± 0.07 vs. 1.33 ± 0.11, p = 0.01, Table 1) and Glx DIFF (4.54 ± 0.40 vs. 3.07 ± 0.26, p = 0.002) in the right frontal lobe compared with female infants. After adjusting for GA at birth and postnatal age (WOL) at ^1^H–MRS, the association of sex remained significant with GABA + (β = 0.27, p = 0.03) and Glx DIFF (β = 1.12, p = 0.01) concentrations. The temporal trend of the metabolite concentrations stratified by sex is depicted in Fig. 4.

### Relationship of GABA + and glutamate concentrations with degree of prematurity at birth

Infants born before 28 weeks of gestation (n = 18), had higher levels of GABA + (1.67 ± 0.07 vs 1.35 ± 0.11, p = 0.01) compared with those born after 28 completed GA weeks (Table 3). The relationship between degree of prematurity and metabolite concentrations remained statistically significant after adjusting for PMA at scan and sex.

**Table 3:** Relationship of right frontal lobe metabolite concentrations with degree of prematurity at birth.

Infant parameters	Infants born < 28 weeks GA (n = 18)	Infants born ≥ 28 weeks GA (n = 20)	p-value^a^
Birth weight (median, IQR)	809 (690, 925)	1169 (1015, 1380)	<0.0001
Small-for-gestation infants (n, %)	0 (0.0)	4 (20.0)	0.11
Male Sex (n, %)	11 (61.1)	8 (40.0)	0.33
African American (n, %)	13 (72.2)	11 (55.0)	0.33
Cesarean Section delivery (n, %)	12 (66.7)	13 (65.0)	1.00
Multiple gestation (n, %)	8 (44.4)	0 (0.0)	0.0009
Apgar at 5 min (median, IQR)	8 (7, 8)	8 (7, 8)	0.17
PMA at ^1^H–MRS in weeks (median, IQR)	38.4 (37.7, 39.4)	38.4 (37.0, 41.4)	0.81
Weeks of life at ^1^H–MRS (median, IQR)	12.6 (11.1, 15.4)	9.3 (7.7, 11.9)	0.0003
Metabolites (mean ± SE in institutional units), accounting for patient clustering			
GABA +	1.67 ± 0.07	1.35 ± 0.11	0.01
Glutamate DIFF	2.89 ± 0.19	2.44 ± 0.20	0.10
Glutamate OFF	3.06 ± 0.31	3.11 ± 0.23	0.88
Glx DIFF	4.57 ± 0.39	3.30 ± 0.29	0.009
Glx OFF	4.38 ± 0.24	4.47 ± 0.29	0.81
NAA	3.86 ± 0.20	3.59 ± 0.29	0.45
Choline	2.10 ± 0.05	1.90 ± 0.06	0.02
Cr	3.68 ± 0.15	3.43 ± 0.15	0.24
Ins	6.96 ± 0.32	6.61 ± 0.28	0.42
GABA +/Cho	0.79 ± 0.03	0.69 ± 0.06	0.12
GABA +/Cr	0.45 ± 0.02	0.39 ± 0.03	0.08
DIFF SNR (median, IQR)	5.5 (4.0, 6.0)	5.0 (4.0, 8.0)	0.90
OFF SNR (median, IQR)	9.0 (7.0, 11.0)	8.0 (7.0, 10.0)	0.38
DIFF FWHM (median, IQR)	0.06 (0.05, 0.07)	0.07 (0.04, 0.09)	0.84
OFF FWHM (median, IQR)	0.04 (0.03, 0.06)	0.05 (0.03, 0.05)	0.62

SNR, signal-to-noise ratio; FWHM, full width at half-maximum.

All metabolite concentrations are in institutional units.

^a^Wilcoxon Mann-Whitney or Fisher’s exact test for demographic and clinical variables; least squares means estimates from generalized estimating equations for metabolite concentration comparisons.

### Correlation of glutamate measurements using OFF and DIFF spectra

**Figure 5:**
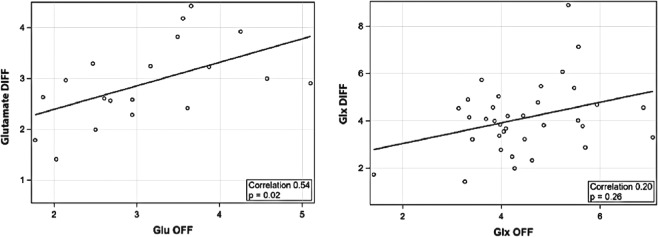
Correlation of glutamate concentrations between OFF and DIFF spectra measurements (**a**) Glutamate and (**b**) Glx.

Glutamate concentrations measured by DIFF spectra moderately correlated with measurements from OFF spectra (Spearman correlation r = 0.54, p = 0.02) (Fig. 5); whereas Glx measurements had low correlation (r = 0.2, p = 0.26). The OFF spectra measurements had significantly higher CRLB for glutamate [glutamate OFF CRLB = 28.9 ± 7.0 vs glutamate DIFF CRLB = 14.6 ± 7.2, p < 0.0001] and Glx [Glx OFF CRLB = 24.0 ± 6.1 vs Glx DIFF CRLB = 11.41 ± 6.8, p < 0.0001].

## Discussion

To the best of our knowledge, this is the first study in very preterm infants to report ex-utero third trimester profiles of *in vivo* GABA + and glutamate concentrations in the frontal lobe. Within the period of 33 to 46 weeks PMA, we observed a significant increase in glutamate concentrations but only a non-significant uptrend in GABA + concentrations. Further, GABA + and Glx (DIFF) concentrations demonstrated a negative correlation with GA at birth and higher concentrations in male infants compared with females.

Our study adds novel data to a limited body of evidence due to the intrinsic technical challenges in GABA and glutamate *in vivo* quantification in the developing brain. GABA concentrations in the developing brain are inherently low (1–2 µmol/g)^19,28,29^ and its spectroscopic signals are overlapped by more dominant metabolites in the standard ^1^H-MRS spectra^15^. To enhance spectral resolution, animal studies have utilized ultra-high strength magnetic field (7–9 T) scanners^30,31^, while adult human studies on 3 T scanners have used alternative techniques including short-echo time and/or spectral editing techniques like MEGA-PRESS^14,32–34^. Additional challenges in the preterm population including smaller brain volumes limiting voxel size, motion artifacts on non-sedated research scans, and dependence on intensive care during the early postnatal period have further limited *in vivo* investigation during this crucial phase of brain development. Only a handful of neonatal ^1^H-MRS studies have reported increasing glutamate concentrations with advancing PMA^17,19,35^. Using MEGA-PRESS spectral editing, a marginal decrease in GABA/Cr between TEA (between 37 to 46 weeks PMA) and 6 months corrected age has been reported in preterm infants (born between 24 to 34 weeks GA)^18^. GABA and glutamate concentrations were noted to be lower in the preterm infant (born <35 weeks GA) brain at TEA compared with healthy term controls and older children (6–16 years age)^17,19^.

We report cross-sectional temporal profiles of neuro-metabolites from the developing right frontal lobe and noted that glutamate concentrations increase with advancing PMA of the infant (Table 2). It is plausible that the degree of brain development (neuronal migration, organization and GABA signal maturation) at the time of preterm birth will have a strong impact on further postnatal extra-uterine brain development. Hence, we further investigated the relationship of metabolite concentrations with the duration of in-utero brain development (represented by gestational age, GA at birth) and extra-uterine development (represented by postnatal age, WOL), which together constitute the corrected PMA. We observed that *in vivo* GABA + , glutamate, NAA and Cr concentrations increase with advancing postnatal age (i.e., WOL, Table 2); perhaps reflecting postnatal neuronal stimulation, organization, synaptogenesis and neurotransmitter signal maturation. However, GABA + (and Glx DIFF) concentrations correlated negatively with increasing GA at birth (Tables 2 & 3), which is a novel finding and warrants further prospective longitudinal investigation. We speculate that preterm birth may arrest/delay the migration of GABA and glutamatergic neurons through the white matter and/or may accelerate neuronal GABA and glutamate production prematurely after early extra-uterine exposure; which may then be captured as increased concentration on ^1^H-MRS in infants born at a lower GA^28^.

NAA and Cr are energy metabolites present in neurons as well as glial cells;^36^ ^1^H-MRS studies in preterm infants have reported increasing NAA, NAA/Cr and NAA/Cho with advancing age and its association with neurodevelopmental outcomes^13,37^. We observed that both NAA and Cr concentrations increased with advancing PMA (Table 2 and Supplement Fig C, likely indicating overall neuronal and glial growth and maturation^19,35,36,38^. Cho (a cell membrane component) and Ins (involved in osmoregulation and cell membrane component) were noted to remain stable with advancing PMA during the early postnatal period, consistent with previous reports (Table 2)^19,35,38^.

We report for the first time that preterm male infants had higher GABA + (and Glx DIFF) concentrations in their frontal lobes than females (Table 1 and Fig. 4). While the observation of sex-based influence is novel in this population, adult studies have reported higher GABA levels in dorsal prefrontal region in males^39^, a faster decline of frontal GABA concentration in aging women^40^, and influence of menstrual and postpartum hormonal changes^41,42^. Further investigation is needed to determine whether this sex specific metabolic profile of the frontal lobe in preterm infants contributes to differential neurodevelopmental outcomes.

GABA and glutamate play a multitude of metabolic, paracrine and regulatory roles in addition to neurotransmission which are vital during fetal and postnatal brain development^43–46^. In fact, GABA is considered to exert an excitatory signal in the fetal brain which transitions to the mature inhibitory signal following a developmentally regulated chloride channel switch during perinatal period^7,47^. It remains unclear whether preterm birth alters this signal transition and disrupts further development of the brain and in particular the frontal lobe, which is a prominent region for GABA and glutamatergic system development during late gestation and plays an important role in cognitive outcomes in preterm infants^48^. Lower levels of brain GABA and glutamate concentrations have been associated with a variety of neurologic disorders including epilepsy, autism and attention deficit disorders^32,49–51^, which are more common in surviving premature infants^3^. Pharmacologic effects of potentially neuroprotective agents like erythropoietin and allopregnanolone as well as injurious agents like benzodiazepines, opioids and anti-seizure drugs administered during intensive care are linked with the GABA and glutamate pathways and hence emphasize the need for further investigation^12,52,53^.

Glutamate concentrations have been previously reported using STEAM, PRESS and MEGA-PRESS; with merits and limitations of each technique^14,16,54–56^. Moderate-to-low correlation between PRESS and MEGA-PRESS measurements reported in adult literature is also observed in our study findings (Fig. 5)^25,54,55^. In fact, it is likely that glutamate (and Glx) measurements by each technique represent different contributions from glutamate and glutamine and/or other signals like macromolecules and baseline differences; and hence each measurement may have clinical relevance based on the particular hypothesis being investigated. On the other hand, it is possible the differences between the DIFF and OFF measurements indicate superiority of one method over the other. In our cohort, we note that the CRLB are significantly lower for DIFF measurements of Glutamate and Glx. We concur that further investigations are needed to identify the ideal measurement technique to measure glutamate (and Glx) and their pathophysiologic relevance in the developing preterm brain.

Although our study is strengthened by the application of ^1^H-MRS MEGA-PRESS, the use of a prospective design and the expertise in acquiring non-sedated MRI in the preterm neonatal population, there are several technical limitations that deserve mention. Technical challenges of acquiring data using time-consuming MEGA-PRESS sequences from the small developing frontal lobe, together with the challenge of motion-artifacts during non-sedated scans may affect the accuracy of the measurements. Although we avoided CSF with meticulous manual placement of the voxels centering on the deep frontal white matter, due to the variable gyrification within the relatively small developing frontal lobe and motion artifact during the ^1^H-MRS acquisition, our voxels likely contain some gray matter (and minimal CSF) contribution. For the current report, the potential effect of differential tissue composition of the voxel on the measured neurometabolites needs to be considered. Tissue segmentation of each voxel is currently being attempted to address some of these challenges. Further, the GABA + measurements from DIFF spectrum contain contribution from macromolecules that co-edit during GABA J-difference experiment but remain unidentified and of uncertain physiologic and developmental significance in the human brain^15^. Current methods for macromolecule correction have limitations; and it is reported that changes in the GABA + signal are primarily driven by changes in GABA concentrations^14,15^. Hence, to be consistent with the conventional literature, we report the measurements as GABA + ^14,15^. Further, the observed temporal relationship of metabolites with advancing PMA is cross-sectional and not based on true longitudinal observations for each infant. Finally, the neurodevelopmental follow-up of the cohort is underway and once completed, will allow further investigation of pathophysiologic significance of *in vivo* GABA and glutamate concentrations measured by ^1^H-MRS.

## Conclusion

This study is the first to describe the relationship of *in vivo* GABA + and glutamate concentrations in the developing frontal lobe with age and sex during the early postnatal period in very preterm infants without structural brain injury. We observed a temporal increase in glutamate concentrations with advancing PMA and in particular, postnatal age but not GA at birth. We also report higher concentrations of GABA + in preterm male infants compared to females. Prospective, longitudinal studies of GABA and glutamate measurements in larger preterm cohorts with long-term neurodevelopmental follow-up are needed to better understand its pathophysiologic and prognostic value in guiding individualized neuroprotective interventions to improve neurodevelopmental outcomes.

## Supplementary information

Supplementary Figures and Table.

## Data Availability

The original datasets generated during and/or analyzed during the current study are available from the corresponding author (C.L.) on reasonable request.
