# Supplementary material for: Age and Sex Influences Gamma-aminobutyric Acid Concentrations in the Developing Brain of Very Premature Infants
Source: Sci Rep. 2020 Jun 29;10:10549. doi: 10.1038/s41598-020-67188-y (PMC7324587; doi:10.1038/s41598-020-67188-y)
Supplement: Supplementary file 1 — Supplementary Figures and Table. [file 41598_2020_67188_MOESM1_ESM.pdf]

Age and Sex Influences Gamma-aminobutyric Acid Concentrations in the Developing Brain of Very Premature Infants

Sudeepta K. Basu MBBS MS[1,2.7], Subechhya Pradhan PhD[2,7], Marni B. Jacobs PhD [3,7], Mariam Said MD[1,2,7], Kushal Kapse MS[2], Jonathan Murnick MD[4,7], Matthew T. Whitehead MD[4,7],Taeun Chang MD[5,7] Adre J. du Plessis MD MBChB[6,7], and Catherine Limperopoulos PhD[2,4,7].

[1]Neonatology, Children's National Hospital, Washington, D.C.

[2]Center for the Developing Brain, Children's National Hospital, Washington, D.C.

[3]Division of Biostatistics and Study Methodology, Children's National Hospital, Washington, D.C.

[4]Division of Diagnostic Imaging and Radiology, Children's National Hospital, Washington, D.C.

[5]Division of Neurology, Children's National Hospital, Washington, D.C.

[6]Fetal Medicine institute, Children's National Hospital, Washington, D.C.

[7]The George Washington University School of Medicine, Washington, D.C.

Supplementary Figure A: Composite superimposed OFF spectrum for all PRESS acquisitions

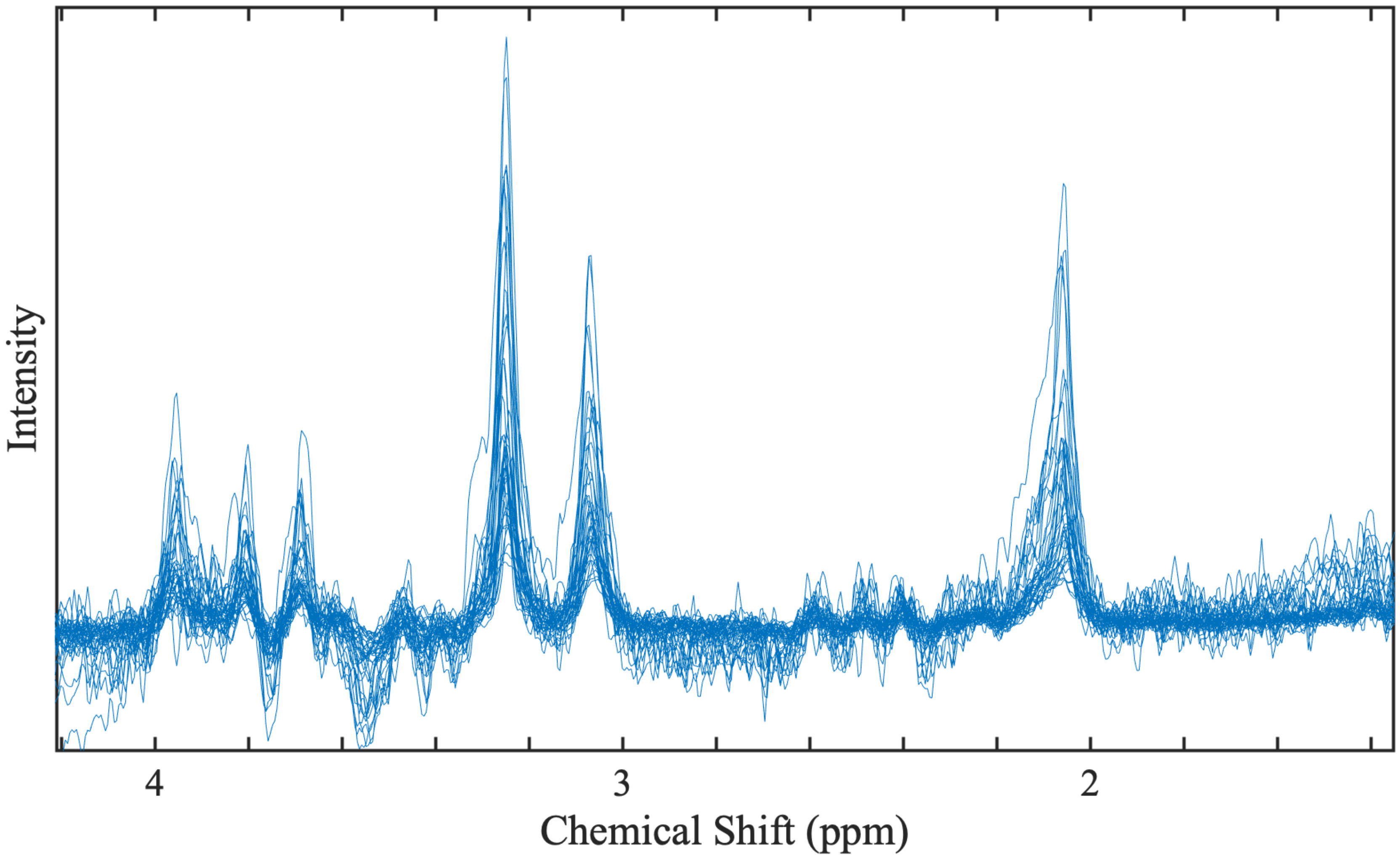

Supplementary Figure B: Composite superimposed DIFF spectrum for all MEGA-PRESS acquisitions

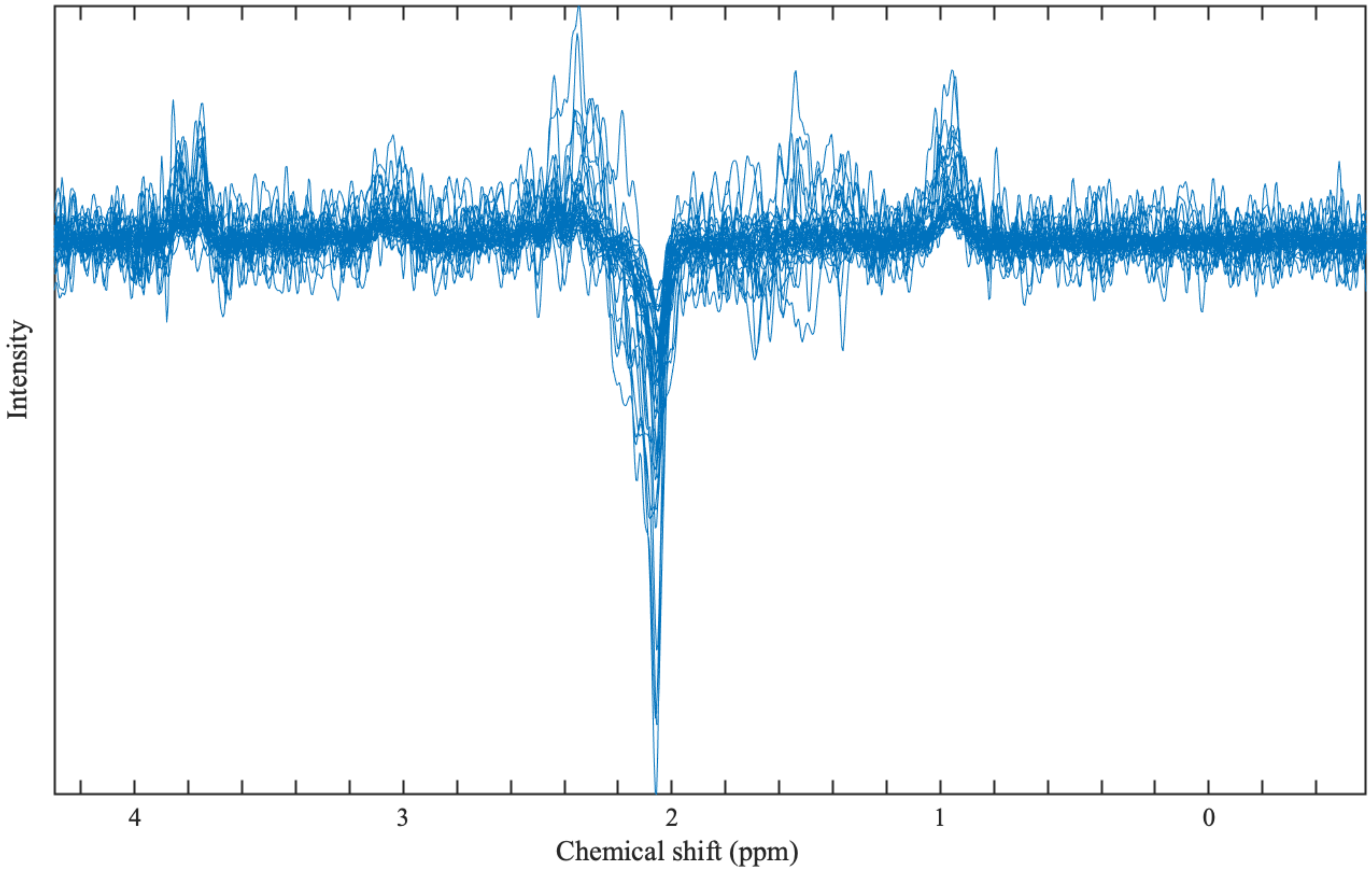

Supplementary Figure C: Metabolite profiles with advancing PMA at MRI: a) NAA, b) Cr and c) Cho

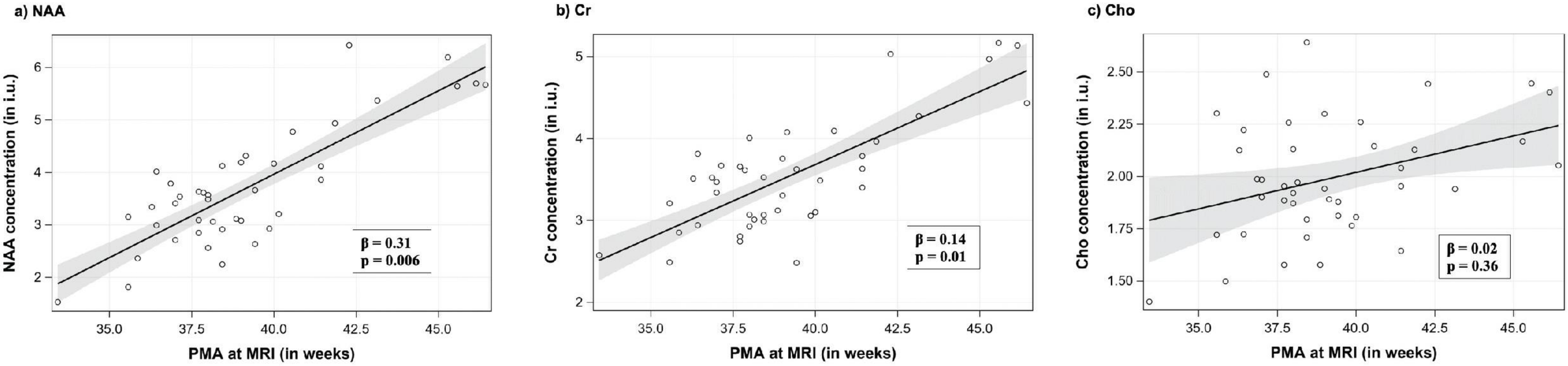

Supplementary Table A. GABA, glutamate and Glx metabolite measurements with a range of Cramer-Rao Lower Bounds (CRLB) inclusion cut-off points

**Metabolites in mean ± SE in institutional units, accounting for patient clustering; (CRLB inclusion cut-off %) (n = number of infants)**	**All infants**	**Male infants**	**Female infants**	**p-value**
GABA (20%) (n = 31)	1.60 ± 0.06	1.70 ± 0.07	1.47 ± 0.09	0.05
GABA (40%, current) (n = 35)	1.50 ± 0.07	1.66 ± 0.07	1.33 ± 0.11	**0.01**
GABA (100%) (n = 35)	Same participants as current analysis			
Glutamate DIFF (20%) (n = 29)	2.82 ± 0.13	2.86 ± 0.18	2.76 ± 0.18	0.69
Glutamate DIFF (40%, current) (n = 33)	2.64 ± 0.15	2.82 ± 0.18	2.44 ± 0.23	0.20
Glutamate DIFF (100%) (n = 35)	2.54 ± 0.16	2.82 ± 0.18	2.25 ± 0.24	0.06
Glutamate OFF (20%) (n = 2)	4.42 ± 0.16	4.26	4.58	**--**
Glutamate OFF (40%, current) (n = 19)	3.09 ± 0.20	3.19 ± 0.24	2.92 ± 0.31	0.50
Glutamate OFF (100%) (n = 34)	2.61 ± 0.16	2.67 ± 0.23	2.53 ± 0.19	0.62
Glx DIFF (20%) (n = 33)	4.05 ± 0.26	4.67 ± 0.38	3.32 ± 0.24	**0.003**
Glx DIFF (40%, current) (n = 36)	3.84 ± 0.27	4.54 ± 0.40	3.07 ± 0.26	**0.002**
Glx DIFF (100%) (n = 36)	Same participants as current analysis			
Glx OFF (20%) (n = 10)	5.58 ± 0.27	4.66 ± 0.25	5.40 ± 0.61	0.70
Glx OFF (40%, current) (n = 33)	4.43 ± 0.19	4.66 ± 0.24	4.18 ± 0.28	0.19
Glx OFF (100%) (n = 38)	4.17 ± 0.20	4.37 ± 0.27	3.96 ± 0.28	0.29
